# The Impact of Frailty and Severe Cognitive Impairment on Survival Time and Time to Initiate Dialysis in Older Adults With Advanced Chronic Kidney Disease: A Prospective Observational Cohort Study

**DOI:** 10.7759/cureus.64303

**Published:** 2024-07-11

**Authors:** Hani Hussien, Lucian Siriteanu, Ionut Nistor, Mehmet Kanbay, Andreea Covic, Luminita Voroneanu, Adrian Covic

**Affiliations:** 1 Department of Nephrology, “Grigore T. Popa” University of Medicine and Pharmacy, Iasi, ROU; 2 Department of Internal Medicine, Koc University School of Medicine, Istanbul, TUR; 3 Department of Nephrology, “Dr. C.I. Parhon” University Hospital, Iasi, ROU

**Keywords:** physical frailty, moca score, ckd elderly, timing of dialysis initiation, cognitive impairment and dementia, pre-dialysis ckd, clinical frailty scale, chronic kidney disease (ckd)

## Abstract

Background and objectives

Frailty and cognitive impairment significantly impact survival time and time to initiate dialysis in older adults with advanced chronic kidney disease (CKD). This study aims to evaluate the effects of frailty and cognitive impairment on these outcomes and determine the most effective assessment tool for predicting early dialysis initiation and short survival time.

Materials and methods

This prospective observational cohort study involved 240 patients aged ≥65 years with stage 4 or 5 CKD, recruited from a nephrology outpatient department (ambulatory care) between March 2020 and March 2021. Frailty was assessed using the Physical Frailty Phenotype (PFP), PRISMA-7, Clinical Frailty Scale (CFS), and FRAIL scale. Cognitive function was evaluated using the Montreal Cognitive Assessment (MoCA). The primary outcomes were time to initiate dialysis and survival time, with secondary outcomes including hospitalization rates, length of stay, and mortality after dialysis initiation.

Results

Frail patients only showed significantly shorter time to dialysis initiation when identified by the PFP and FRAIL scale (28.3 months for frail vs. 31.2 months for non-frail, p = 0.028; 26.9 months for frail vs. 30.9 months for non-frail, p = 0.038). The PFP, FRAIL, and CFS tools indicated significantly shorter survival times for frail patients compared to non-frail patients (26.8 months for frail vs. 30.6 months for non-frail, p = 0.003). Frailty is strongly correlated with severe cognitive impairment, as 45.5% of frail patients (according to the FRAIL scale) have dementia compared to 15.1% of non-frail patients (p<0.001). However, cognitive impairment did not significantly affect the time to dialysis initiation or survival time. Hospitalization rates and length of stay in the hospital were significantly higher only for frail patients identified by PRISMA-7, with a median hospital length of stay of 9.15 days for frail patients vs 6.37 days for non-frail patients (p = 0.044). Overall, 37.5% of the patients did not survive during the study follow-up, with frail patients having a higher mortality rate.

Conclusion

Frailty, mainly when assessed by PFP and FRAIL, is a significant predictor of early dialysis initiation and reduced survival time in older adults with advanced CKD. Cognitive impairment, while prevalent, did not independently predict these outcomes. Regular frailty screening should be incorporated into CKD management to tailor interventions and improve patient outcomes.

## Introduction

Chronic kidney disease (CKD) is a progressive disorder marked by a gradual decline in kidney function as time passes [1]. CKD impacts around 10% of the world's population, with a more significant occurrence among elderly individuals [2,3]. The rising incidence of CKD in older individuals is caused by numerous factors, including population ageing, the coexistence of various comorbidities, such as diabetes mellitus and arterial hypertension, and the recent advancements in the treatment of these conditions, which have resulted in increased survival rates among older patients [2,3]. It is widely recognised that individuals experience a steady fall in the kidney filtration rate, referred to as kidney senescence, after the age of 40, and this decline occurs at a rate of around 1% per year [4]. The decline in the glomerular filtration rate (GFR) is continuous, and the GFR can drop below 45 mL/min in many people by age 65 [5]. Previous research has indicated that approximately 11% of individuals who are 65 years old and do not have any significant additional health conditions have stage 3 or higher CKD [5]. Hypertension and type 2 diabetes mellitus are the primary risk factors for CKD, impacting more than 50% of individuals aged 65 and above, frequently coexisting simultaneously [6]. In addition, the process of ageing is linked to nutrient malabsorption, obesity resulting from a sedentary lifestyle, and frailty, all of which together contribute to the progression of CKD [7-9]

Frailty is defined as a decline in physiological reserves and an increased susceptibility to minor stressors, resulting in poor health outcomes such as reduced quality of life and increased mortality [10]. Frailty is more common in older adults with CKD, making its management more difficult and leading to poor prognosis. Frailty in patients with CKD has been linked to disease progression, increased hospitalisation rates, hospital length of stay (LOS) and raised mortality [11]. Although frailty diagnosis is essential, there is currently no widely agreed upon single tool for identifying and detecting frailty in the general population or older adults with CKD. A recent comprehensive analysis found that there are 91 different tools utilised in 140 studies to assess frailty, indicating the wide range and intricate nature of frailty evaluation [7]. There are two well-known models for evaluating frailty: the Fried Frailty Phenotype model, which focuses on physical characteristics, and the cumulative deficit approach, also known as the frailty index (FI) [12-14]. Although both models efficiently detect frailty, they have practical difficulties due to their complex nature and time demands, making them impractical for daily practice [15]. Although commonly used, the Physical Frailty Phenotype (PFP) can be a time-consuming approach that may not comprehensively consider the multifactorial aspects of frailty. Additional instruments, such as the PRISMA-7 questionnaire [16] and the CFS are frequently employed; however, their sensitivity and specificity differ [7,17,18]

Managing CKD in older people presents difficulties due to the complex nature of the disease and the coexistence of comorbidities. Historically, the primary approach to managing kidney disease has been renal replacement therapy (RRT), such as dialysis. However, there has been an increasing focus on conservative kidney management (CKM) or comprehensive conservative care [19]. CKM is especially applicable to frail, geriatric, or terminally sick patients who may not derive substantial advantages from dialysis. CKM encompasses strategies aimed at decelerating the progression of the disease, actively managing symptoms, engaging in shared decision-making, and providing psychological and social assistance [20]. The primary objective of CKM is to enhance the overall quality of life and minimise the frequency of hospitalisations and the severity of symptoms [19,20]. Research has shown that CKM can provide similar rates of survival and quality of life as dialysis in older persons with advanced CKD, especially those who are over 80 years old or have substantial comorbidities [21-25]. The choice between RRT and CKM should be determined based on the patient's general health condition, personal preferences, and considerations for their quality of life. Recently proposed guidelines and research have recommended opting for CKM in frail older adults [26,27]. Hence, it is crucial to use a frailty tool that best detects frailty with low false positive cases and can predict a shorter time to initiate dialysis and short survival time or low survival expectancy; therefore, CKM can be started if the patient has opted for it. Similarly, CKD is a risk factor for cognitive impairment, and interestingly, cognitive impairment contributes to poor outcomes in patients with CKD [7,10].

To the best of our knowledge, this is the first study in older adults with advanced CKD that uses different frailty assessment tools to assess the correlation between frailty and severe cognitive impairment on one side and a shorter time to initiate dialysis as well as survival time on the other side. The objective of this study is to evaluate the impact of frailty, as diagnosed using different frailty tools, and cognitive impairment on the duration it takes for older adults with advanced CKD to start dialysis and their overall survival time. Additionally, to define the best frailty diagnostic tool that better predicts a shorter time to initiate dialysis and short survival time. The findings from this study will provide valuable information for developing individualised and proper management for this vulnerable population.

## Materials and methods

Study design

This prospective observational cohort study is designed to evaluate the impact of frailty, as assessed by the Frailty Phenotype, PRISMA-7, CFS, and FRAIL Scale, and cognitive impairment, evaluated using the Montreal Cognitive Assessment (MoCA) test, on time to dialysis initiation and survival time in older adults with advanced CKD. The study was approved by the Ethical Committee of Dr. C.I. Parhon University Hospital (Nr 8044/18.10.2019)

Patients aged ≥65 years with stage 4 or 5 CKD (estimated glomerular filtration rate ≤29 mL/min/1.73m^2^, calculated using the Chronic Kidney Disease Epidemiology Collaboration formula) were recruited from the nephrology outpatient department of Dr. C.I. Parhon University Hospital in Iasi city between March 2020 and March 2021. Patients were randomly selected for study participation in a cluster sampling manner, with the clusters being the specific doctors on different days, to prevent sampling bias. The exclusion criteria included dialysis dependence, history of kidney transplantation, recent acute kidney injury, initial visit with the provider, amputation, blindness, a recent cerebrovascular accident in the last three months before enrollment, or having vascular access for dialysis. Patients were followed prospectively until starting long-term maintenance dialysis, death, or for a maximum of three years from the date of enrollment. Survival time and time to dialysis initiation were the primary outcomes, with secondary outcomes being the frequency of hospitalisations, LOS, and survival time after starting maintenance dialysis.

Measurement of baseline characteristics and outcomes

Baseline data encompassed diverse patient characteristics identified by previous studies as influential on the primary outcome of survival rate and time to dialysis initiation. These included patient demographics, medical history, polypharmacy (the concurrent use of five or more different drugs), baseline pathology measurements, measures of functional status, and factors that may contribute to social disparities in health, such as living conditions, income, educational level, and family living status.

Frailty was evaluated using the Physical Frailty Phenotype (PFP), PRISMA-7, CFS, and FRAIL Scales. The PFP and FRAIL Scale have a score range of 0-5, where a score of 0 indicates robust health, scores of 1-2 signify pre-frailty, and scores of 3-5 indicate frailty. The PRISMA-7 is a seven-item questionnaire that solicits binary responses (yes or no) to gather information about demographics (age and gender), physical capabilities, the presence of medical conditions that restrict the individual, and any dependency on others. The scoring ranges from 0 to 7, where a higher score corresponds to greater frailty severity-a score of 3 or above signals the necessity for additional evaluation. The PRISMA-7 can be completed in under five minutes by the individual or a well-informed representative about their typical health condition, and it can be used in various settings, including the community, emergency units, outpatients, and in-patient departments. The CFS integrates visual and textual clinical data and assigns scores ranging from one (indicating robust health) to nine (denoting terminal illness). The scale designates a score of four as suggestive of 'vulnerable' (pre-frail), and a score of five or above is recognised as indicative of a state of 'living with frailty.'. We have used the FFP as the gold-standard tool because it is the most cited tool in the literature for frailty assessment.

Cognitive function was evaluated using the MoCA test, which scored out of 30. A score of 25 to 18 signifies mild cognitive impairment, while a score of 17 or less indicates dementia, the test developer's proposed cut-off for dementia. The primary outcomes were time to initiate dialysis and time to death, while the secondary outcomes were prevalence of frailty, all-cause mortality, incidence of dialysis, number of hospitalisations, LOS, and mortality after starting maintenance dialysis.

Statistical analysis

Statistical analyses were performed using IBM SPSS Statistics for Windows, Version 25 (Released 2017; IBM Corp., Armonk, New York, United States). All analyses were two-tailed, and a p-value of <0.05 was considered statistically significant. Descriptive statistics for continuous variables were presented as mean ± standard deviation (SD) or median (IQR) after assessing normality with the Shapiro-Wilk test, while categorical variables were summarised with frequencies and percentages. Comparative analysis between frail and non-frail patients was performed per frailty assessment tool using t-tests or Mann-Whitney U tests for continuous variables and Chi-squared or Fisher's exact tests for categorical variables. The Kaplan-Meier method and Cox proportional-hazards regression analyses, with variables p<0.10 in the univariate analysis included in the multivariate model, were employed for survival time and time to dialysis initiation, with the proportional-hazards assumption checked using Schoenfeld residuals. Correlations among frailty tools, baseline characteristics, and cognitive and functional scores were evaluated using Spearman or Pearson coefficients. Multivariate logistic regression identified frailty and cognitive impairment predictors and adjusted for confounders significant in univariate analysis.

## Results

General characteristics

Two hundred forty patients were included in this study between March 2019 and 2020 and were followed up for three years. Table 1 provides an overview of the demographic and clinical characteristics of the study cohort. The median age of the patients was 73 years, with 129 (53.8%) female patients and 111 (46.3%) male patients. The median number of comorbidities was 5, with a median Charlson Comorbidity index score of 5 points, indicating a moderate comorbidity burden. Notably, 61 (25.4%) of patients had a 10-year expected survival rate of 0%, while 51 (21.3%) had a survival rate of 53%, and 16 (6.7%) had a survival rate of 90% or more. Weight loss was reported in 69 (28.7%) of cases. The median estimated glomerular filtration rate (eGFR) was 20. Finally, 90 (37.5%) of patients did not survive during the study period, as shown in Table 1.

**Table 1:** Descriptive characteristics of the analysed patients IQR: Interquartile Range; CCI: Charlson Comorbidity Index; eGFR: Estimated Glomerular Filtration Rate

Parameter	Value
Age (Median (IQR))	73 (68-79)
Male Gender (Nr., %)	111 (46.3%)
Female Gender (Nr., %)	129 (53.8%)
Education < 4 Classes (Nr., %)	46 (19.2%)
Education 5-8 Classes (Nr., %)	61 (25.4%)
Education 9-12 Classes (Nr., %)	82 (34.2%)
Superior Education (Nr., %)	51 (21.3%)
Living Single (Nr., %)	68 (28.3%)
Living with a Family (Nr., %)	172 (71.7%)
Income ≤ 200$, (Nr., %)	40 (16.7%)
Income > 200$ (Nr., %)	200 (83.3%)
Urban Location (Nr., %)	152 (63.3%)
Rural Location (Nr., %)	88 (36.7%)
Alcohol Consumption (Nr., %)	49 (20.4%)
Smoking (Nr., %)	36 (15%)
Ex-smoking (Nr., %)	28 (11.7%)
Comorbidities number (Median (IQR))	5 (4-6)
CCI Points (Median (IQR))	5 (4-7)
Weight Loss (Nr., %)	69 (28.7%)
Polypharmacy (Nr., %)	197 (82.1%)
Anaemia (Nr., %)	163 (67.9%)
Dialysis Initiation (Nr., %)	60 (25%)
eGFR in mL/min/1.73m^2^ (Median (IQR))	20 (15-24.75)
Death (Nr., %)	90 (37.5%)

**Figure 1:**
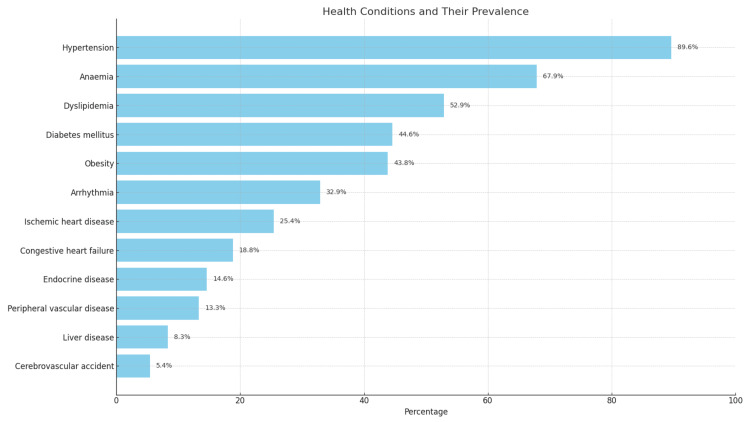
Distribution of the patients according to comorbidities

The most frequent comorbidities among the patients were hypertension, affecting 215 patients (89.6%), followed by anaemia in 163 patients (67.9%) and dyslipidemia in 127 patients (52.9%). Less frequent comorbidities included cerebrovascular accidents in 13 patients (5.4%), liver disease in 20 patients (8.3%), and peripheral vascular disease in 32 patients (13.3%), as shown in Figure 1.

Frailty

Table 2 presents the prevalence of frailty among the patients. Frailty was assessed using four distinct tools, revealing variations in frailty identification. The PFP identified 98 patients (40.8%) as frail, while PRISMA-7, CFS, and FRAIL scale generated frailty rates of 165 patients (68.8%), 104 patients (43.3%), and 55 patients (22.9%), respectively. Additionally, 90 patients (37.5%) had a slow gait speed, and 126 (52.5%) had weak handgrip strength.

**Table 2:** Distribution of the patients according to frailty status

Frailty Tool	Value (Nr., %)
Frailty Phenotype	
0 points	38 (15.8%)
1 point	47 (19.6%)
2 points	57 (23.8%)
3 points	41 (17.1%)
4 points	35 (14.6%)
5 points	22 (9.2%)
Robust	38 (15.8%)
Pre-frail	104 (43.3%)
Frail	98 (40.8%)
FRAIL Scale	
0 points	16 (6.7%)
1 point	116 (48.3%)
2 points	53 (22.1%)
3 points	32 (13.3%)
4 points	21 (8.8%)
5 points	2 (0.8%)
Robust	16 (6.7%)
Pre-frail	169 (70.4%)
Frail	55 (22.9%)
PRISMA-7 Scale	
0 points	2 (0.8%)
1 point	22 (9.2%)
2 points	51 (21.3%)
3 points	68 (28.3%)
4 points	45 (18.8%)
5 points	43 (17.9%)
6 points	9 (3.8%)
No risk of Frailty	75 (31.3%)
At risk of frailty	165 (68.8%)
Clinical Frailty Scale	
Managing well	25 (10.4%)
Vulnerable	111 (46.3%)
Mild	61 (25.4%)
Moderate	32 (13.3%)
Severe	11 (4.6%)

When we used the PFP as a diagnosis reference,the FRAIL scale had a sensitivity of 54.08%, a specificity of 98.59%, and an accuracy of 80.42% for the diagnosis of frailty, while frailty using the PRISMA-7 scale had a sensitivity of 89.8%, a specificity of 45.77%, and an accuracy of 63.75% for the diagnosis of frailty. Finally, frailty using the CFS has a sensitivity of 86.73%, a specificity of 86.62%, and an accuracy of 86.67% for the diagnosis of frailty, as shown in Table 3.

**Table 3:** Comparison of the frailty tools to the PFP as the reference tool *Fisher's Exact Test CFS: Clinical Frailty Scale; PFP: Physical Frailty Phenotype; Statistically Significant p<0.05

Frailty (Phenotype) / Frailty (FRAIL)	Non-frail		Frail		p*
	Nr.	%	Nr.	%	
Non-frail (FRAIL)	140	98.6%	45	45.9%	<0.001
Frail (FRAIL)	2	1.4%	53	54.1%	
Frailty (Phenotype) / Frailty (PRISMA-7)	Non-frail		Frail		p*
	Nr.	%	Nr.	%	
Non-frail (PRISMA-7)	65	45.8%	10	10.2%	<0.001
Frail (PRISMA-7)	77	54.2%	88	89.8%	
Frailty (Phenotype) / Frailty (CFS)	Non-frail		Frail		p*
	Nr.	%	Nr.	%	
Non-frail (CFS)	123	86.6%	13	13.3%	<0.001
Frail (CFS)	19	13.4%	85	86.7%	

The results show that PRISMA-7 is the most sensitive tool for detecting frailty and that the FRAIL scale is the most specific, while CFS has a balance in sensitivity and specificity.

Time-to-initiate dialysis

The average mean for time-to-initiate dialysis was 30 months (95% confidence interval (CI): 28.58-31.43), while the average survival time was 29 months (95% CI: 27.69-30.33), as shown in Table 4.

**Table 4:** Time-to-initiate dialysis and survival time CI: Confidence Interval

Parameter	Average	Standard Error	95% CI
Time-to-Initiate Dialysis (TID)	30	0.727	28.58-31.43
Survival Time	29	0.673	27.69-30.33

In our study, the TID was significantly different only among patients categorised as frail by the PFP (p = 0.028) and the FRAIL scale (p=0.038). Specifically, frail patients, as defined by the PFP, had an average TID of 28.259 months (95% CI = 25.720-30.718) compared to 31.151 months for non-frail patients (95% CI = 29.478-32.823). Similarly, frail patients on the FRAIL scale-initiated dialysis in an average of 26.874 months (95% CI = 23.275-30.473), while non-frail patients took 30.874 months (95% CI = 29.377-32.372). Nevertheless, when patients were classified based on the PRISMA-7 or CFS, the difference in the time-to-initiate dialysis was not statistically significant (p = 0.121 and p = 0.365, respectively), as shown in Table 5.

**Table 5:** Time-to-initiate dialysis comparison between frailty groups CFS: Clinical Frailty Scale; CI: Confidence Interval; Statistically Significant at p<0.05

Frailty Assessment	Group	Average in Months	Standard Error	95% CI	p*
Phenotype	Non-frail	31.151	0.853	29.478 - 32.823	0.028
	Frail	28.219	1.275	25.720 - 30.718	
FRAIL Scale	Non-frail	30.874	0.764	29.377 - 32.372	0.038
	Frail	26.874	1.836	23.275 - 30.473	
PRISMA-7 Scale	Non-frail	31.038	1.318	28.455 - 33.620	0.121
	Frail	29.553	0.865	27.857 - 31.249	
CFS	Non-frail	30.537	0.92	28.734 - 32.340	0.365
	Frail	29.29	1.171	26.994 - 31.585	

Table 6 shows the Cox proportional hazard models for predicting dialysis according to frailty status. The univariate analyses demonstrated that frailty detected by the PFP was associated with a 1.752-fold increase in the risk of dialysis initiation (95% CI: 1.054-2.91, p=0.031). Similarly, frailty measured by the FRAIL scale corresponded to a 1.803-fold higher risk (95% CI: 1.025-3.172, p=0.041). However, these associations were not statistically significant in the multivariate model (p>0.05).

**Table 6:** Cox proportional hazard models for predicting haemodialysis according to frailty status *Likelihood Ratio Test; CI: Confidence Interval; Statistically Significant p<0.05

Parameter (Frailty)	Univariate		Multivariate (p=0.057*)	
	OR (95% C.I.)	P	OR (95% C.I.)	p
Phenotype	1.752 (1.054-2.91)	0.031 (p=0.028*)	1.504 (0.794-2.847)	0.210
FRAIL	1.803 (1.025-3.172)	0.041 (p=0.038*)	1.349 (0.664-2.741)	0.408

Life expectancy and survival time

The study participants were classified according to their Charlson Comorbidity Index (CCI score), predicting a 10-year survival expectancy. The cohort had a median CCI score of 5 points, with an interquartile range (IQR) of 4 to 7. The CCI points were analysed with the expected 10-year survival probabilities, as shown in Table 7.

**Table 7:** Charlson comorbidity index in the cohort IQR: Interquartile Range; CCI: Charlson Comorbidity Index

10-Year Survival Expectancy According to the CCI	Number (%)
0%	61 (25.4%)
2%	37 (15.4%)
21%	42 (17.5%)
53%	51 (21.3%)
77%	33 (13.8%)
90%	16 (6.7%)

Patients with very low survival expectancy (0-2% 10-year survival) included 61 (25.4%) of the cohort with a CCI of 0%, while patients with a CCI of 2% formed 37 (15.4%) of the whole cohort. Meanwhile, the percentage of patients with moderate survival expectancy (21-53%) included 42 (17.5%) of the cohort with a CCI of 21% and 51 (21.3%) with a 10-year survival expectancy of 53%. Among the study population, patients with a high survival expectancy (77-90% 10-year survival) were less: those with a CCI of 77% accounted for 33 (13.8%), while those with a CCI of 90% survival expectancy over the next 10 years made up only 16 (6.7%).

**Figure 2:**
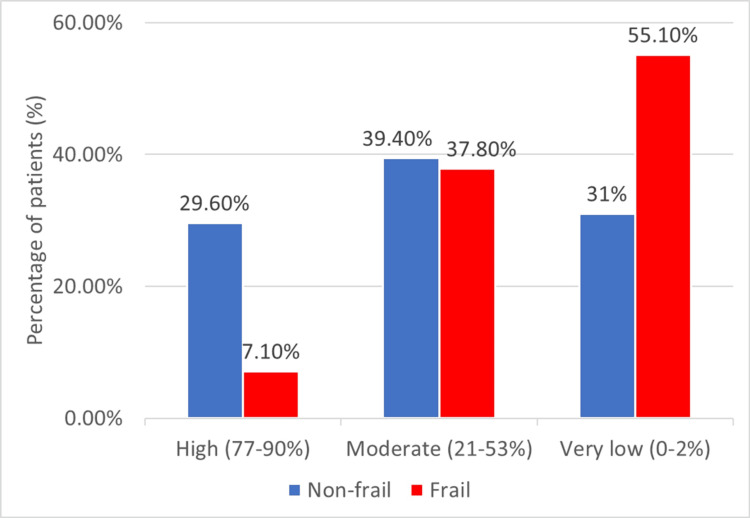
Distribution of the patients according to the Charlson comorbidity index– 10-year survival expectancy and frailty (phenotype)

As shown above, most of the study cohort had very low or moderate 10-year survival expectancy. The patients were then divided based on the CCI to determine their 10-year survival expectancy and were categorized by their frailty status. The data revealed significant differences in survival expectancy between frail and non-frail patients evaluated using PFP as the gold standard (Figure 2).

Patients with high 10-year survival expectancy (77-90%) had a much lower rate of frailty, with only seven frail patients (7.1%) compared to 42 non-frail patients (29.6%). Patients with moderate 10-year survival expectancy (21-53%) had a relatively equal distribution between frail and non-frail patients, with 56 non-frail patients (39.4%) and 37 frail patients (37.8%). Patients with very low 10-year survival expectancy (0-2%) had a greater prevalence of frailty. Frail patients accounted for 54 (55.1%) of the total, while non-frail patients comprised 44 (31%). Statistical analysis indicated that these differences were significant (p<0.001).

Regarding time to death, the results show that patients with frailty classified by PFP (p=0.003), FRAIL scale (p=0.001) and CFS (p=0.041) but not the PRISMA scale (p=0.918) had a significant difference in the survival time (Table 8). In a comparative survival analysis, patients identified as frail by those three tools exhibited differing average survival times. Specifically, frail patients classified by the phenotype tool had an average survival time of 26.750 months (95% CI = 24.586-28.915), which was shorter compared to the 30.571 months for non-frail counterparts (95% CI = 28.965-32.177).

**Table 8:** Survival time comparison between the frailty groups CFS: Clinical Frailty Scale; CI: Confidence Interval; Statistically Significant at p<0.05

Frailty Assessment	Group	Average Time in Months	Standard Error	95% CI	p*
Phenotype	Non-frail	30.571	0.82	28.965 - 32.177	0.003
	Frail	26.75	1.104	24.586 - 28.915	
FRAIL Scale	Non-frail	30.215	0.719	28.805 - 31.625	0.001
	Frail	24.961	1.542	21.938 - 27.984	
PRISMA-7 Scale	Non-frail	29.215	1.237	26.791 - 31.639	0.918
	Frail	28.918	0.801	27.437 - 30.489	
CFS	Non-frail	30.262	0.848	28.600 - 31.923	0.041
	Frail	27.375	1.067	25.285 - 29.466	

Similarly, those considered frail according to the FRAIL scale had an even lower average survival time of 24.961 months (95% CI = 21.938-27.984) versus 30.215 months for non-frail individuals (95% CI = 28.805-31.625). Lastly, frailty, as assessed by the CFS, was associated with an average survival of 27.375 months (95% CI = 25.285-29.466), compared to 30.262 months for those not identified as frail (95% CI = 28.600-31.923). These findings consistently indicate shorter survival times in frail patients across all scales (Table 8).

Data from Table 9 shows the Cox proportional hazard models for predicting mortality according to frailty status. In the univariate analysis, the risk of mortality was significantly increased in frail individuals when assessed by different scales. Frailty, as defined by the phenotype model, resulted in a 1.837-fold increase in mortality risk (95% CI: 1.214-2.779, p=0.004). According to the FRAIL scale, frail patients had a higher risk, at a 2.087-fold increase (95% CI: 1.345-3.238). Furthermore, the CFS indicated a 1.535-fold increased risk of death (95% CI: 1.015-2.322, p=0.041). However, in the multivariate model that considered all variables simultaneously, none of the frailty measures were significant predictors of mortality (p>0.05).

**Table 9:** Cox proportional hazard models for predicting mortality according to frailty status *Likelihood Ratio Test; CI: Confidence Interval; Statistically Significant at p<0.05

Parameter (Frailty)	Univariate		Multivariate (p=0.006*)	
	OR (95% C.I.)	P	OR (95% C.I.)	p
Phenotype	1.837 (1.214-2.779)	0.004 (p=0.003*)	1.443 (0.731-2.848)	0.289
FRAIL	2.087 (1.345-3.238)	0.001 (p=0.001*)	1.651 (0.934-2.919)	0.084
CFS	1.535 (1.015-2.322)	0.042 (p=0.041*)	0.957 (0.524-1.748)	0.887

Moreover, during the three years of follow-up, 18 patients (7.5%) of the total studied cohort underwent dialysis and subsequently died. Within this subgroup, 11 patients (61.1%) were frail according to the phenotype assessment, and six patients (33.3%) had dementia. The average time to initiate dialysis was 11.55 ± 7.1 months, while the survival time was 19.84 ± 6.62 months, as seen in Table 10.

**Table 10:** Assessment of cognitive function using the Montreal Cognitive Assessment (MoCA) test Normal (26 ≥); Mild Cognitive Impairment (25 to 18); Dementia < 18

Cognitive function	(Nr., %)
Normal cognitive function	38 (15.8%)
Mild cognitive impairment	149 (62.1%)
Dementia	53 (22.1%)

Cognitive function impact of frailty, TID and survival time

In the study, patients underwent the MoCA with a median score of 21, falling within an interquartile range of 18 to 24.75. When categorized based on their cognitive function, 38 patients (15.8%) were classified as normal, 149 patients (62.1%) exhibited mild cognitive impairment, and 53 patients (22.1%) were diagnosed with dementia, meaning that almost 85% of the study population had a degree of cognitive impairment (Table 10).

**Figure 3:**
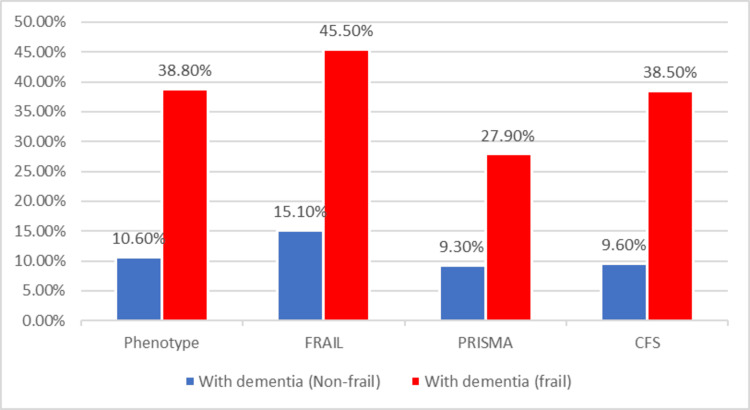
Distribution of the patients according to frailty status and existence of dementia CFS: Clinical Frailty Scale

Patients with severe cognitive impairment (MOCA score of 17 or less) had a significantly strong association with frailty degree across the four assessment tools when compared to patients without dementia. The prevalence of dementia in frail patients versus non-frail patients was significant across all scales (p < 0.001). According to the PFP, dementia was present in 38 frail patients (38.8%) compared to 15 non-frail patients (10.6%). The FRAIL scale showed that 55 frail patients (45.5%) had dementia, whereas only 28 non-frail patients (15.1%) did. Using the PRISMA-7 scale, 46 frail patients (27.9%) were diagnosed with dementia, in contrast to seven non-frail patients (9.3%). Similarly, the CFS indicated that 52 frail patients (38.5%) had dementia, compared to 13 non-frail patients (9.6%), as displayed in Figure 3.

The odds ratios (OR) indicate substantially higher odds of frailty among those with dementia, with the Phenotype scale showing an OR of 5.362 (95% CI = 2.739-10.499), the FRAIL scale an OR of 4.673 (95% CI = 2.401-9.904), the PRISMA-7 scale an OR of 3.755 (95% CI = 1.606-8.778), and the CFS presenting an OR of 5.913 (95% CI = 2.952-11.847) (Figure 3).

Nevertheless, patients with dementia did not show significant differences in time to initiation of dialysis (p=0.563) or overall survival time (p=0.197) when compared with patients without dementia (normal cognitive function or mild cognitive impairment).

Number of hospitalisations and LOS

The differences in the number of hospitalisations between all frailty groups were not statistically significant (p>0.05) according to the Mann-Whitney U tests, indicating no significant difference between frail and non-frail patients. Similarly, the LOS between almost all frailty groups was not statistically significant (p>0.05), except for the PRISMA 7 - Frailty groups, where patients at risk of frailty had a significantly higher LOS (median = 3, IQR = 0-14) compared to patients without risk (median = 0, IQR = 0-11) (p=0.044) (Table 11).

**Table 11:** Comparison of the number of hospitalisations between the frailty groups *Mann-Whitney U Test; CFS: Clinical Frailty Scale; SD: Standard Deviation; IQR: Interquartile Range; Statistically Significant at p<0.05.

Measure	Group	Average ± SD	Median (IQR)	Average Rank	p*
No. Hospitalisations/Phenotype	Non-frail	0.8 ± 1.19	0 (0-1)	114.93	0.102
	Frail	1.02 ± 1.26	1 (0-2)	128.58	
No. Hospitalisations/FRAIL	Non-frail	0.85 ± 1.21	0 (0-1)	118.67	0.413
	Frail	1 ± 1.29	1 (0-1)	126.66	
No. Hospitalisations/PRISMA-7	Non-frail	0.72 ± 1.22	0 (0-1)	108.77	0.054
	Frail	0.96 ± 1.22	1 (0-1)	125.83	
No. Hospitalisations/CFS	Non-frail	0.82 ± 1.2	0 (0-1)	116.58	0.275
	Frail	0.98 ± 1.26	1 (0-2)	125.63	

Similarly, the LOS between almost all frailty groups was not statistically significant (p>0.05), except for the PRISMA 7 - Frailty groups, where patients at risk of frailty had a significantly higher LOS (median = 3, IQR = 0-14) compared to patients without risk (median = 0, IQR = 0-11) (p=0.044), as shown in Table 12.

**Table 12:** Comparison of the length of stay between the existence of frailty groups *Mann-Whitney U Test; CFS: Clinical Frailty Scale; LOS: Length of Stay; SD: Standard Deviation; IQR: Interquartile Range; Statistically Significant at p<0.05.

Measure	Group	Average ± SD	Median (IQR)	Average Rank	p*
LOS/Phenotype	Non-frail	7.66 ± 11.47	0 (0-13)	116.09	0.203
	Frail	9.17 ± 11.86	4.5 (0-14)	126.89	
LOS /FRAIL	Non-frail	7.95 ± 11.5	0 (0-13)	118.72	0.435
	Frail	9.38 ± 12.1	4 (0-17)	126.47	
LOS /PRISMA-7	Non-frail	6.37 ± 10.81	0 (0-11)	108.04	0.044
	Frail	9.15 ± 11.91	3 (0-14)	126.16	
LOS /CFS	Non-frail	8.08 ± 11.76	0 (0-13)	118.43	0.571
	Frail	8.54 ± 11.5	2 (0-13.75)	123.20	

Additionally, differences in the number of hospitalisations and LOS between cognitive function groups or dementia groups were not statistically significant (p>0.05), and there was no significant correlation between the number of hospitalisations and MoCA score (p=0.922 and p=0.781, respectively).

Finally, the differences in the number of hospitalisations and LOS between mortality groups were not statistically significant (p>0.05). However, for dialysis groups, patients who started dialysis had a significantly higher number of hospitalisations (median = 1.5, IQR = 1-3 vs median = 0, IQR = 0-1, p<0.001) and LOS (median = 16.5, IQR = 13-28 vs median = 0, IQR = 0-6, p<0.001) compared to patients without dialysis as shown in Table 13. 

**Table 13:** Comparison of the number of hospitalisations and length of stay between the existence of dialysis and mortality *Mann-Whitney U Test; IQR: Interquartile Range; SD: Standard Deviation; Statistically Significant at p<0.05.

Measure	Group	Average ± SD	Median (IQR)	Average Rank	p*
No. hospitalisations/Dialysis	Absent	0.53 ± 1	0 (0-1)	99.36	<0.001
	Present	1.97 ± 1.23	1.5 (1-3)	183.92	
No. hospitalisations/Death	Absent	0.87 ± 1.24	0 (0-1)	118.22	0.473
	Present	0.92 ± 1.2	1 (0-1)	124.31	
Length of Stay/Dialysis	Absent	4.39 ± 8.73	0 (0-6)	96.99	<0.001
	Present	19.95 ± 11.5	16.5 (13-28)	191.03	
Length of Stay/Death	Absent	8.19 ± 11.92	0 (0-13)	118.75	0.587
	Present	8.42 ± 11.18	3.5 (0-13)	123.42	

## Discussion

This study provides significant insights into the impact of frailty and cognitive impairment on the length of survival and the time required to initiate dialysis in older adults diagnosed with CKD. The findings underscore the importance of early detection of frailty and the need for individualised treatments to improve patient outcomes. According to our study, frailty significantly shortened the time it took to start dialysis when measured using the PFP and the FRAIL scale. Also, the previous studies did not evaluate frailty during the advanced stages of CKD but at the time of dialysis initiation or using one tool for frailty screening [28,29]. Similarly, it was proposed that frail patients had a shorter duration before initiating dialysis than non-frail patients [30], and our study has confirmed this hypothesis. The data from our study suggest that frailty can accelerate the progression of CKD and the requirement for RRT. Nevertheless, the PRISMA-7 and CFS did not show a statistically significant variation in the time it took to start dialysis, which makes them unsuitable for frailty diagnosis but only suitable for screening. The strong connection between frailty and the need to initiate maintenance dialysis early shows how important it is to include screening and diagnosis for frailty in regular medical exams and CKD guidelines since frailty can be reversed in its early stages. Moreover, the study revealed that frailty substantially affects the duration of survival. Patients who were categorised as frail based on the PFP, FRAIL, and CFS tools had shorter survival times compared to non-frail patients. The study also found that frailty is a strong predictor of mortality in older adults diagnosed with advanced CKD or even in those who have started chronic dialysis. Patients with frailty showed a higher risk for death, emphasising the urgent necessity for fast detection of frailty in order to decide which management modality we would follow. Additionally, the four frailty assessment tools demonstrated a strong correlation between the presence of frailty and cognitive impairment, particularly in cases of severe cognitive impairment (dementia). Individuals who have dementia have a higher degree of frailty in comparison to those who do not have severe cognitive impairment. However, cognitive impairment did not have a significant impact on the time it took to start maintenance dialysis or the overall length of survival. These findings indicate that while cognitive impairment is prevalent in older persons with advanced CKD, it may not be a significant predictor of dialysis initiation or mortality compared to frailty in this particular population. Also, our study found no notable differences in the number of hospitalisations or LOS between frail people and those who were not, except when frailty was assessed using the PRISMA-7 tool. Patients identified as being at risk of frailty, as assessed using the PRISMA-7, had longer LOS in the hospital, possibly because the method is highly sensitive in identifying adults at risk of frailty, and it is intuitive that older adults would show some elements of frailty when they are exposed to an acute event that has led to their hospital admission. Nevertheless, the lack of notable differences in other tools implies that frailty alone may not be the critical factor influencing hospitalisations in older patients diagnosed with CKD.

Limitations of the study

This study is subject to several limitations. Due to its observational nature, establishing a cause-and-effect relationship based solely on the observed associations is not possible. The study was conducted at a single centre, which may limit the generalisability of the findings to a broader population, particularly regarding the number of hospitalisations. Additionally, the use of self-reported questionnaires introduces the potential for recall bias, especially among participants with severe cognitive impairment.

## Conclusions

This study emphasises the importance of frailty diagnosis, especially in older adults with advanced CKD. The physical frailty phenotype and FRAIL tools effectively identified older patients at a higher risk of early dialysis initiation and experiencing decreased survival time. Moreover, frail patients had a significant prevalence of cognitive impairment; however, cognitive impairment did not independently impact the time to initiate dialysis or survival time.

These findings provide evidence for adopting routine frailty screening in CKD management to tailor therapeutic interventions and improve patient outcomes. Future research should evaluate the incorporation of frailty and cognitive assessments into the medical care plans of older adults diagnosed with CKD.
